# Toward Accurate Spin–Orbit Splittings from Relativistic Multireference Electronic Structure Theory

**DOI:** 10.1021/acs.jpclett.4c01372

**Published:** 2024-07-02

**Authors:** Zijun Zhao, Francesco A. Evangelista

**Affiliations:** Department of Chemistry and Cherry Emerson Center for Scientific Computation, Emory University, Atlanta, Georgia 30322, United States

## Abstract

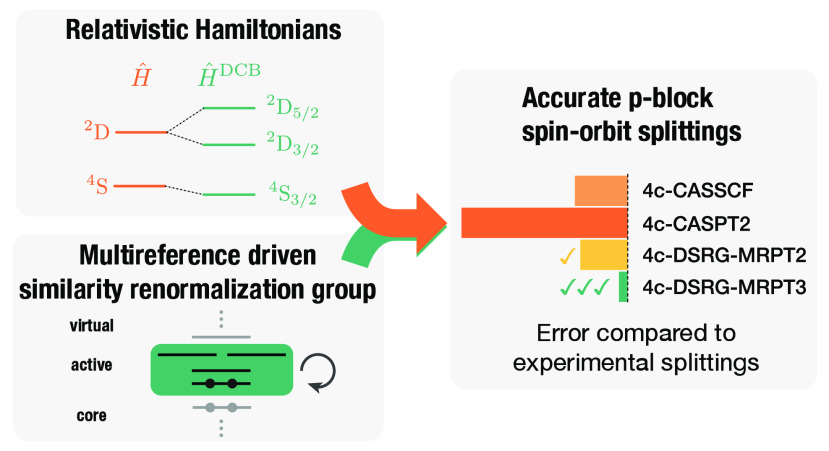

Most nonrelativistic electron correlation methods can be adapted to account for relativistic effects, as long as the relativistic molecular spinor integrals are available, from either a four-, two-, or one-component mean-field calculation. However, relativistic multireference correlation methods remain a relatively unexplored area, with mixed evidence regarding the improvements brought by perturbative treatments. We report, for the first time, the implementation of state-averaged four-component relativistic multireference perturbation theories to second and third order based on the driven similarity renormalization group (DSRG). With our methods, named 4c-SA-DSRG-MRPT2 and 3, we find that the dynamical correlation included on top of 4c-CASSCF references can significantly improve the spin–orbit splittings in p-block elements and potential energy surfaces when compared to 4c-CASSCF and 4c-CASPT2 results. We further show that 4c-DSRG-MRPT2 and 3 are applicable to these systems over a wide range of the flow parameter, with systematic improvement from second to third order in terms of both improved error statistics and reduced sensitivity with respect to the flow parameter.

The accurate description of the electronic structure of systems containing heavy atoms requires a balanced treatment of both strong electron correlation and relativistic effects.^1,2^ The former arises from the commonplace occurrence of near-degenerate configurations in such systems, and the latter arise from the *Z*^2^ dependence of relativistic contributions to the valence electron energy,^3^ where *Z* is the atomic number. The effects of spin–orbit coupling (SOC), which are completely absent from the nonrelativistic electronic Hamiltonian, are reflected in spectroscopic measurements,^4−6^ have an outsized influence on reaction energetics^7,8^ and NMR shielding constants,^9^ and play an important role in photophysics and photochemistry.^10−12^ Four-component (4c) mean-field and electron correlation methods employ Hamiltonians that explicitly couple the spin and orbital angular momentum degrees of freedom, and are the methods of choice to treat SOC effects in an *ab initio* manner. However, relativistic electron correlation methods are not always able to consistently improve the description of SOC effects from relativistic mean-field references.^13^

Computational methods for treating strongly correlated systems have been an area of intense study.^14−33^ Most nonrelativistic correlation methods can be readily adapted to account for relativistic effects within the no-pair approximation, as long as the complex-valued molecular spinor integrals are available,^34−36^ after either a two- or four-component mean-field calculation. As such, considerable work has been devoted to developing mean-field methods. On the four-component side, the Dirac–Hartree–Fock (DHF, or 4c-HF) and complete active space self-consistent field (4c-CASSCF)^37−40^ can be routinely carried out in several packages.^41−46^ Depending on whether a 2*c*/4c-HF or CASSCF reference is used, these methods are further grouped into single-^35,47^ or multireference approaches.^48−52^ Multireference relativistic methods have received more attention than their nonrelativistic cousins, as many systems where a relativistic study is warranted, such as late-row transition metal, lanthanide, and actinide complexes, exhibit strong configuration mixing in the ground state, and as a result require the zeroth order wave function to be multiconfigurational. Examples of relativistic multireference theories include Fock-space multireference coupled cluster (FSMRCC),^1^ 4c internally contracted MRCI (ic-MRCI), CASPT2,^2^ NEVPT2,^53^ generalized van Vleck PT2 (GVVPT2),^54^ multireference Møller–Plesset (MRMP).^1,55^ In several ways, these methods reflect one or more limitations of their nonrelativistic counterparts: (1) lack of proper scaling with system size, (2) difficulties with scaling to large active spaces, (3) numerical divergences arising from the intruder state problem, and (4) insufficient accuracy in the description of dynamical electron correlation.

To address the above-mentioned insufficiencies of relativistic multireference theories, we have explored a 4c generalization of the multireference driven similarity renormalization group (MR-DSRG) approach.^56,57^ An overview of this approach is given in Figure 1. The MR-DSRG is a size-extensive, low-scaling, numerically robust, and systematically improvable many-body formalism for treating dynamical electron correlation starting from strongly correlated reference states.^56,57^ In this paper, we show that a combination of 4c strongly correlated reference states and the MR-DSRG is a very promising route to systematically account for relativistic and correlation effects. We demonstrate this point by reporting the first implementation of 4c MR-DSRG methods truncated to second- and third-order in perturbation theory (4c-DSRG-MRPT2/3) and benchmarking them on the spin–orbit splittings of second- to fourth-row atoms, which previous work has shown to be a weak point for four-component correlation methods.^13^

**Figure 1:**
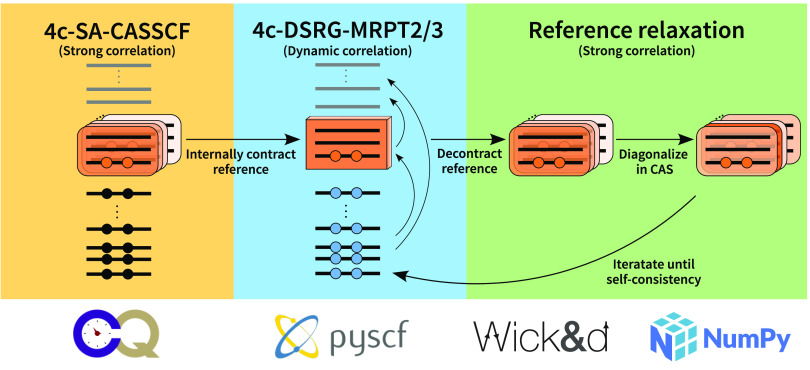
Summary of the 4c-SA-DSRG-MRPT2/3 algorithm. The iterative reference relaxation process is terminated once a threshold is reached.

The starting point for formulating any relativistic quantum chemical theory is the first-quantized relativistic many-electron Hamiltonian:^58^
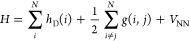
1where *h*_D_(*i*) is the one-electron Dirac Hamiltonian for electron *i*, and *g*(*i*, *j*) is the two-electron interaction operator for an electron pair *i* and *j*. The latter can contain either the bare-Coulomb operator, leading to the Dirac–Coulomb (DC) Hamiltonian, or can additionally include the Gaunt and gauge terms, leading to the Dirac–Coulomb–Gaunt (DCG) or Dirac–Coulomb–Breit (DCB) Hamiltonians. These operators are defined as
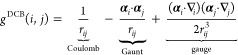
2where **α**_*i*_ is a 3-vector of the Dirac matrices for particle *i*. The Coulomb operator contains charge–charge and spin-same-orbit interactions, the Gaunt and Breit operators contain additional current–current and spin-other-orbit interactions, and a gauge term resulting from working in the Coulomb gauge.^34,36^ Following common practice, we use the DCB Hamiltonian in combination with the no-pair approximation, whereby the many-body basis is constructed from positive-energy spinors only.^58^ The four-component molecular spinors {ψ_*i*_(***r***)} are expanded in a two-spinor basis set {ϕ_μ_^*S*/*L*^} for the small/large components. To avoid variational collapse, we impose the kinetic balance condition, 2*c*ϕ_μ_^*S*^ = **σ**·***p***ϕ_μ_^*L*^, where **σ** is a 3-vector of Pauli matrices.^59^ The kinetic balance condition was imposed at the integral level, using the libcint integral library.^60^

We consider the most general, state-averaged formulation of the MR-DSRG approach, which can target one or multiple electronic states in one computation. We approximate the *k*-th electronic state to zeroth-order with 4c-CASSCF states of the form
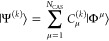
3where *N*_CAS_ is the number of CAS determinants. In 4c-CASSCF, the spinor orbitals {ψ_*i*_} that enter into Slater determinants {Φ^μ^} and the complex CASCI coefficients *C*_μ_^(*k*)^ are simultaneously optimized via the minimax principle.^61^ No positronic orbitals enter into the determinants, in what is known as the “no dressed pair” or “no virtual pair” approximation (NVPA).^40,62^ Furthermore, to ensure the correct degeneracies of the electronic states, we use the state-averaged CASSCF formalism (4c-SA-CASSCF),^63^ where the average energy of a chosen set of states is optimized with respect to the variational parameters.^64^

To account for dynamical electron correlation, we combine 4c-(SA-)CASSCF wave functions with the multireference driven similarity renormalization group (MR-DSRG) formalism.^56^ Having obtained the 4c-(SA)-CASSCF molecular spinor orbitals, the 4c-MR-DSRG theory can be formulated using the normal-ordered second-quantized version of the relativistic no-pair Hamiltonian in eq 1:
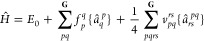
4The orbital indexing convention and definition of orbital spaces used in this work reflects the partitioning of the orbitals into core, active, and virtual sets, as shown in Figure 2. The quantities *f*_*p*_^*q*^ that enter in the Hamiltonian are elements of the generalized Fock matrix, *f*_*p*_^*q*^ = *h*_*p*_^*q*^ + ∑_*ij*_^**H**^*v*_*pi*_^*qj*^γ_*j*_^*i*^, where γ_*j*_^*i*^ is the one-body density matrix defined for a general state Ψ as γ_*j*_^*i*^ = ⟨Ψ|*â*_*i*_^†^*â*_*j*_|Ψ⟩. The MR-DSRG performs a continuous unitary transformation of the Hamiltonian:
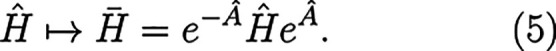
5The operator *Â* is anti-Hermitian, and is expressed in terms of a cluster operator as *Â* = *T̂* – *T̂*^†^. *T̂* is parameterized as in the internally contracted generalization of coupled-cluster theory^65−67^ as *T̂* = *T̂*_1_ + ... *T̂*_*n*_, where a general *k*-body term is expressed as
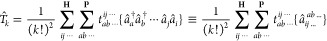
6where {·} indicates normal-ordering with respect to a correlated reference state Ψ or ensemble density.^68^

**Figure 2:**
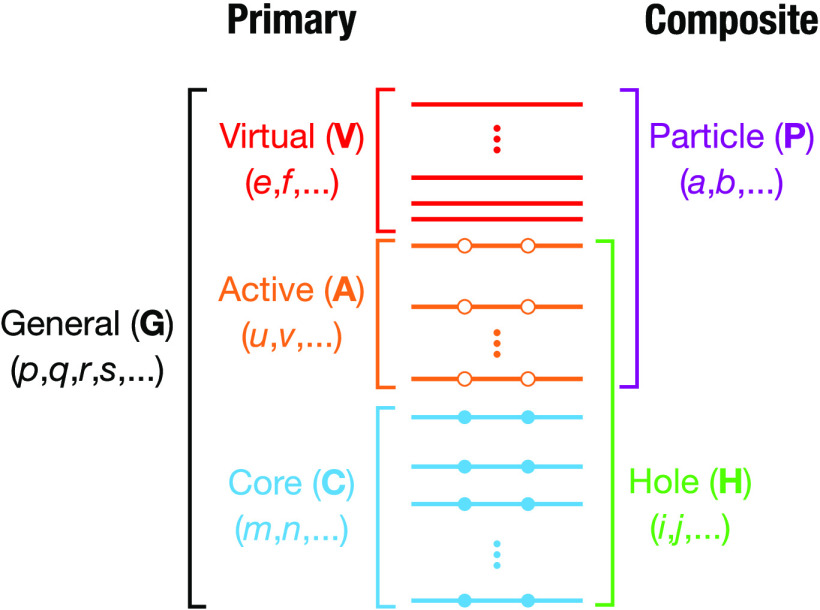
Partitioning of the orbital basis, and the orbital indices used in the MR-DSRG formalism. Note that all positronic orbitals have been excluded as discussed above.

The effect of the MR-DSRG transformation is to bring the Hamiltonian into a more band-diagonal form by zeroing the couplings between the multiconfigurational reference |Ψ_0_⟩ and the excited configurations, i.e., the set of internally contracted determinants |Ψ_*ij*..._^*ab*···^⟩ = {*â*_*ij*..._^*ab*···^}|Ψ_0_⟩.^68,69^ To avoid numerical divergences caused by intruder states, in the DSRG we solve a regularized equation that enforces the decoupling of the off-diagonal elements of *H̅*, denoted as [*H̅*]_od_. This equation reads as [*H̅*(*s*)]_od_ = *R̂*(*s*), where *R̂*(*s*) is a regularization term derived by a second-order perturbative analysis of the similarity renormalization group.^56,70,71^ Several authors have pointed out the benefits of introducing a parameterized denominator shift or a regularizer of the first-order amplitudes in second-order perturbation theory, both in its single and multireference versions.^72,73^ In the DSRG, this role is played by the flow parameter *s*, which in typical applications is found to be optimal for values in the range *s* ∈ [0.35, 2] *E*_h_^–2^, depending on the truncation level.^74^ To generate equations and corresponding tensor contractions for the 4c relativistic extension of the MR-DSRG truncated to second- and third-order in perturbation theory, we have developed the automated code implementation pipeline depicted in Figure 3, which is available in an online code repository.^75^ This approach enables the rapid implementation of highly complex electronic structure theories, removing the potential for human error. This workflow uses our code generator Wick&d(76) coupled to relativistic integrals obtained from PySCF.^44,45,60^ Since 4c-CASSCF is currently not available in PySCF, we generated the corresponding spinor coefficients using the ChronusQ package.^43^

**Figure 3:**
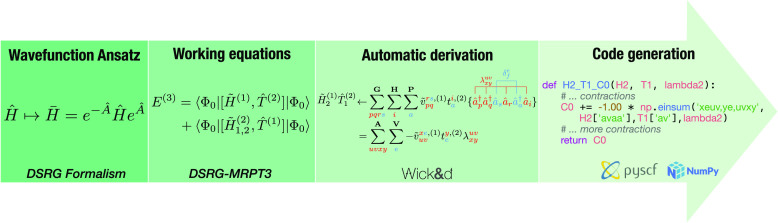
Overview of the automated strategy used in this paper to implement the 4c DSRG methods. Starting from the left, the DSRG ansatz is perturbatively expanded to give working equations for the energy corrections. These are then turned into tensor contractions using the open-source Wick&d package.^76^ Contractions generated by Wick&d are then automatically compiled into executable Python code.

To examine the ability of the 4c-DSRG-MRPT2/3 to improve systematically upon 4c-CASSCF, we computed spin–orbit splittings of second- to fourth-row p-block elements^13^ and the spectroscopic constants for the hydroxyl radical. The p-block splittings span three orders of magnitude, from a few reciprocal centimeters (cm^–1^) to a few thousand, as shown in Figure 4. Therefore, they form a good set of benchmarks for both the absolute and relative accuracies of relativistic methods. To enable direct comparison with the work of Zhang et al.,^13^ we employ the uncontracted correlation-consistent polarized valence triple-ζ (uc-cc-pVTZ) basis set.^80−82^ While this basis is not optimized for 4c relativistic computations, for the systems under consideration, it yields spin splittings within 5 cm^–1^ from a basis set of the same quality optimized for 4c computations.^13^ Further computational details are found in Section 1 of the Supporting Information.

**Figure 4:**
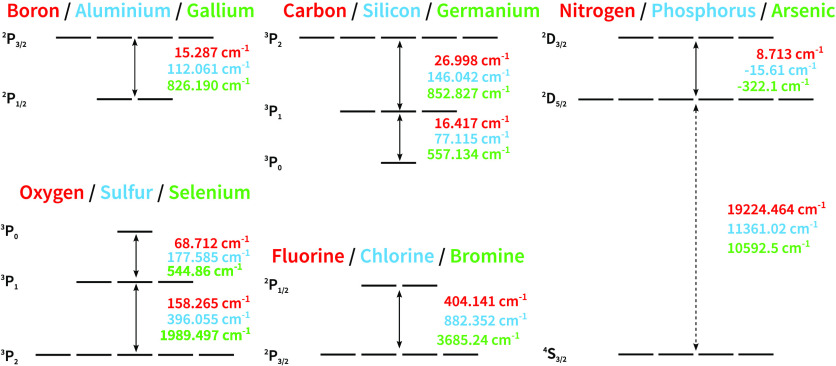
Scheme of the spin–orbit splittings of the p-block elements discussed. State-averaging was performed over all states shown. The splittings denoted with a dotted line are excluded from this analysis and are only included in the state-averaging procedure to ensure variational stability. A switch between the ^2^D_3/2_ and ^2^D_5/2_ states in the group 15 elements is indicated by negative splittings. Energy levels were accessed from the NIST Atomic Spectra Database.^77^

In Figure 5, we compare error statistics for the splittings for the 15 p-block elements calculated with 4c-CASSCF, 4c-SA-DSRG-MRPT2/3, 4c-CASPT2, and 4c-MRCISD+Q (taken from the work by Zhang et al.^13^) to the experimental splittings for three values of the flow parameter (0.2, 0.35, and 0.5 *E*_h_^–2^), which overlap with the range of flow parameters used in nonrelativistic computations and produce results competitive with 4c CASSCF and CASPT2. Table S1 reports splittings for each state with optimal flow parameters that achieve the smallest mean absolute error in cm^–1^ for the whole set of elements for each of the methods respectively (0.24 *E*_h_^–2^ for MRPT2 and 0.35 *E*_h_^–2^ for MRPT3). Looking at the distribution of errors (top plot in Figure 5), both MRPT2 and 3 achieve better average performance on the test set of atoms than CASSCF and the two other multireference electron correlation methods (except for *s* = 0.5 *E*_h_^–2^ in MRPT2). Improving the treatment of dynamical electron correlation from second to third order results in a systematic reduction of the error in the splittings and a decrease in flow parameter sensitivity. Across all methods, the main outliers in percentage error (bottom plot in Figure 5) are elements with small (10–150 cm^–1^) splittings, with the exceptions of arsenic (322 cm^–1^). The complete data set for the splittings can be found in Sections 2 and 3 in the Supporting Information. When compared according to signed errors, irrespective of the value of *s*, the main outliers are fourth-row elements with large splittings, with selenium and gallium being the worst offenders. We also note that DSRG-MRPT3 achieves subwavenumber accuracy for boron and carbon, and rather impressively, less than 2 cm^–1^ errors for the fourth-row selenium and bromine. 4c-CASPT2 and 4c-MR-CISD+Q can be seen to consistently and severely underestimate spin–orbit splittings. This behavior was also pointed out by Zhang et al.^13^ to occur for smaller and larger basis sets than the ones used here. In Table S2, we report additional splittings represented by dashed arrows in Figure 4. We again observe that both MRPT2 and 3 outperform CASPT2, which in turn outperforms CASSCF in this set of transitions. This shows that the 4c-SA-DSRG formalism is capable of accurately capturing SOC effects in higher excited states.

**Figure 5:**
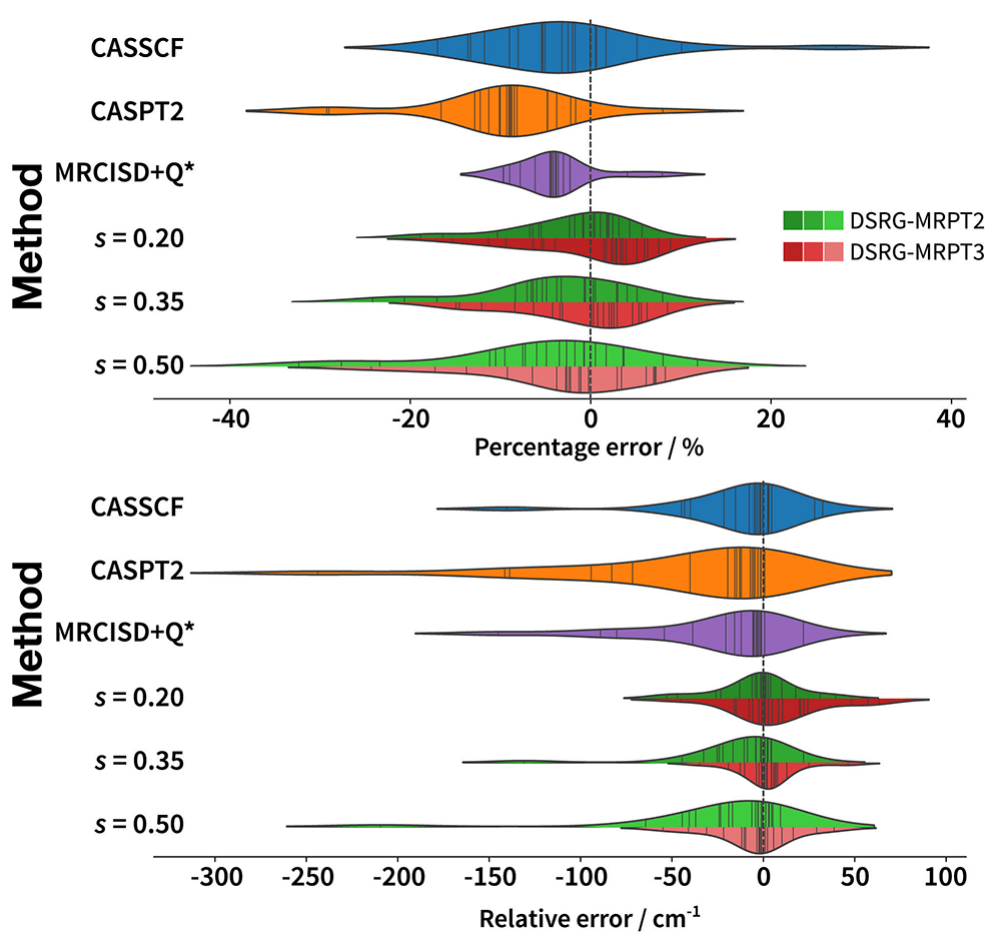
Distributions of the errors in the computed spin–orbit splittings. The dashed vertical lines at 0 represent experimental splittings. The plots were generated with the violinplot function of seaborn,^78,79^ where the data points and the kernel density estimates of the splitting errors of each method are shown. As the most error-prone data points were intractable for MRCI, it is not appropriate to compare it directly with other methods.

After assessing the quality of DSRG-based methods, we turn to the question of the dependence of our results on the flow parameter and its optimal choice for spin–orbit coupling computations. To this end, we studied the variation of the mean absolute percentage error in the splittings as the flow parameter is varied. This quantity is plotted in Figure 6 for both the DSRG-MRPT2/3. In the middle inset, we first observe the correct limiting behavior for *s* = 0, where MRPT2 and MRPT3 reduce to CASSCF. For both PT2 and PT3, the mean absolute percentage errors are smaller than the CASSCF and CASPT2 values for a large range of *s*, up to *s* ≈ 0.75 *E*_h_^–2^ for PT3. For PT2, and PT3 especially, these values overlap with the commonly used range *s* ∈ [0.35, 2.0] *E*_h_^–2^. The kinks in the main curves are artifacts of the absolute values, as the mean signed error curves (bottom insets) are smooth. These results show that the *error profiles* of MRPT2 and MRPT3 do not simply interpolate between those of CASSCF and CASPT2 and bring systematic improvements from a balanced treatment of dynamical correlation.

**Figure 6:**
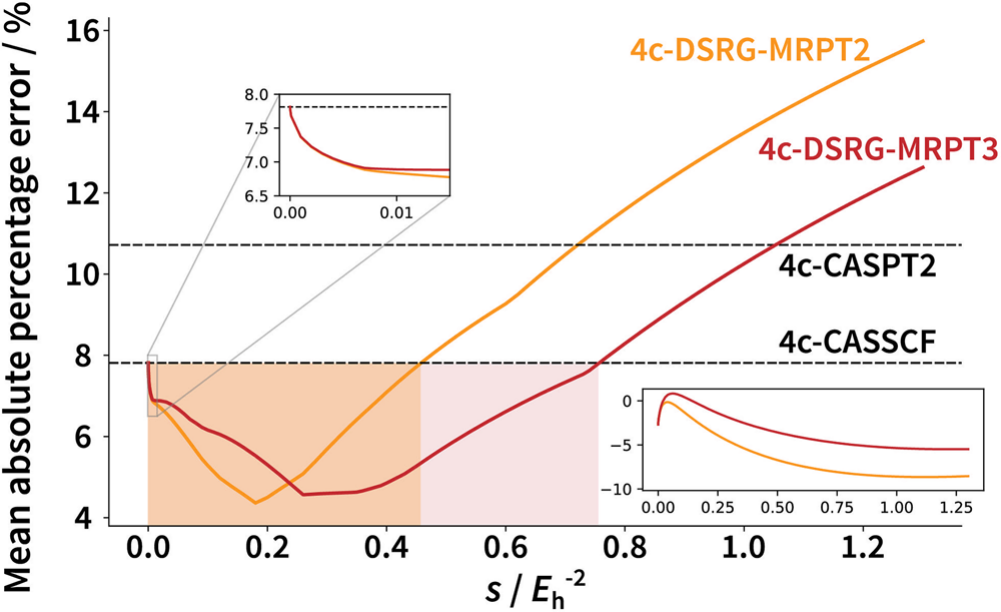
Variation of the mean average error of 4c-DSRG-MRPT2 and MRPT3 as a function of the flow parameter, *s*, for all of the splittings computed using the DCB Hamiltonian with DCB CASSCF molecular spinor coefficients. The main curves show absolute errors and the bottom insets show signed errors. The middle insets zoom into the region close to *s* = 0.

Sections 5 and 6 in the Supporting Information report summary error statistics broken down into groups and periods. An analysis of this data shows that second-row elements display significantly larger absolute and percentage errors, which cannot be simply attributed to the elements having smaller splittings. This is most likely due to the poor description of the core region by the relatively small uncontracted cc-pVTZ basis set for boron through fluorine. As SOC effects decay as 1/*r*^3^,^83^ having variational flexibility in the core region is crucial: Visscher and Dyall^84^ found that the cc-pVTZ basis augmented with an additional tight p function reduced the error in the spin–orbit splitting of F_2_ 10-fold to 1%. We have tested this hypothesis by following Visscher and Dyall and added a tight p function with the exponent of 250.83491 to the cc-pVTZ basis of fluorine. The resulting ^2^P_3/2_ → ^2^P_1/2_ splitting error of fluorine for MRPT3 using *s* = 0.35 *E*_h_^–2^ improved from −12.46 cm^–1^ (−3.1%) to −1.04 cm^–1^ (−0.3%). Analogous data for the remaining second-row elements are reported in Section 7 of the Supporting Information. From these results, we can see that except for the N ^2^D_5/2_ → ^2^D_3/2_ splitting, the provision of more core flexibility significantly improves the description of second-row valence SOC effects with a negligible increase in basis size. The improvement in the underlying CASSCF splittings largely drives the improved description. However, DSRG-MRPT2 and 3 still provide an improvement over the CASSCF results. Another aspect we investigated is a reduction of the cost of the 4c-CASSCF procedure, which in our computations, especially on third- and fourth-row elements, is overwhelmingly the most time-consuming step. In Section 8 of the Supporting Information, we report additional data for a mixed scheme aimed at reducing this cost that uses a combination of the DC and DCB Hamiltonians in the 4c-CASSCF and DSRG-MRPT2 computations. The data presented therein supports the use of such a mixed scheme, as the reduction in accuracy is negligible. The effects of reference relaxation are examined in Section 9 of the Supporting Information, and we found that reference relaxation is able to improve the accuracy of the splittings compared to experimental values.

Lastly, we consider the accuracy of potential energy surfaces computed with the 4c-DSRG-MRPT methods. For example, in Table 1 we report the spectroscopic constants of the ground state of the hydroxyl radical computed with 4c-CASSCF and 4c-DSRG-MRPT3. In this molecule, SOC results in a zero-field splitting (ZFS) of the ground X ^2^Π state into two states with Ω = 3/2 and 1/2 respectively.^85−87^ Our results show that although both 4c-CASSCF and 4c-DSRG-MRPT predict ZFSs in excellent agreement with the experimental value,^88^ the spectroscopic constants from the 4c-DSRG-MRPT3 potential display significantly smaller errors. This point is particularly evident for the harmonic vibrational frequency of the OH radical, which 4c-CASSCF underestimates by 210.9 cm^–1^ vs 8.7 cm^–1^ for the 4c-DSRG-MRPT3. Finally, we computed the potential energy surfaces of the X ^2^Π_3/2_ and ^2^Π_1/2_ states.

**Table 1:** Spectroscopic Constants of the X ^2^Π Ground State of the OH Radical Calculated with Different Methods^a^

method	Δ*r*_*e*_ (pm)	Δω_*e*_ (cm^–1^)	Δω_*e*_*x*_*e*_ (cm^–1^)	Δ*D*_0_ (eV)	ΔZFS (cm^–1^)
4c-SA-CASSCF	0.337	–210.9	30.2	–0.914	–5.2
4c-SA-PT2	0.472	–71.3	–20.5	–0.174	–6.7
4c-SA-PT3	–0.112	–8.7	10.1	–0.224	–4.0
exp	96.966	3737.8	84.9	4.392	139.2

a Shown are the differences with respect to experimental values, taken from the Diatomic Molecular Spectroscopy Database.^88,89^ The (adiabatic) zero-field splitting includes anharmonic zero-point vibrational energy contributions.

In summary, we have presented second- and third-order four-component multireference perturbation theories based on the driven similarity renormalization group formalism, 4c-DSRG-MRPT2/3. We benchmarked these methods on the spin–orbit splittings of second- to fourth-row p-block atoms, showing that they outperform both the underlying 4c-CASSCF and other four-component electron correlation methods, namely, 4c-MR-CISD+Q and 4c-CASPT2. We have further shown that 4c-DSRG-MRPT2 and 3 are applicable to these systems over a wide range of the flow parameter, with systematic improvements in error metrics and sensitivity with respect to *s* from second to third order. In our calculations, most of the wall time is spent in the integral transformation step, which is known to be the main drawback of four-component theories. Exact two-component (X2C) theories with atomic mean-field spin–orbit effects (amfX2C)^90−93^ are an attractive way to reduce the computational effort not only of integral transformations but also in the SCF iterations themselves, depending on the flavor of X2C used. In the future, we plan to explore the combination of amfX2C with two-component MR-DSRG methods to extend multireference relativistic computations to larger systems. Overall, these preliminary results show that a combination of relativistic Hamiltonians and multireference DSRG methods could open many new avenues in modeling the chemistry of open-shell species containing transition metals and heavy elements.

## Data Availability

The data that support the findings of this study are available within the article, its Supporting Information, and from the corresponding author upon reasonable request. The code that can reproduce the data presented within this work is available in an accompanying public GitHub repository.^75^

## Supporting Information Available

Computational details; Tables S1–S9; supporting references (PDF)
