# Supplementary material for: Toward Accurate Spin–Orbit Splittings from Relativistic Multireference Electronic Structure Theory
Source: J Phys Chem Lett. 2024 Jul 2;15(28):7103–10. doi: 10.1021/acs.jpclett.4c01372 (PMC11261625; doi:10.1021/acs.jpclett.4c01372)
Supplement: Supplementary file 1 — jz4c01372_si_001.pdf [file jz4c01372_si_001.pdf]

Supplementary information: “Towards Accurate Spin-Orbit Splittings from Relativistic Multireference Electronic Structure Theory”

Zijun Zhao[1, a)] and Francesco A. Evangelista[1, b)]

Department of Chemistry and Cherry Emerson Center for Scientific Computation, Emory University, Atlanta, Georgia 30322, USA

a)Electronic mail: zijun.zhao@emory.edu

b)Electronic mail: francesco.evangelista@emory.edu

I. COMPUTATIONAL DETAILS

We used the Dirac–Coulomb–Breit (DCB) Hamiltonian with the uncontracted Dunning's correlation-consistent polarized valence triple-zeta (uc-cc-pVTZ) basis set,[1–3] to enable direct comparison with the work of Zhang *et al.*[4] Finite Gaussian nuclei were used,[5] and non-valence orbitals in all third- and fourth-row atoms from the correlated calculations were frozen.

For the p-block elements, whenever the neutral atom cannot be converged by the 4c-HF procedure, the corresponding ion with the configuration of the closest noble gas is used instead (*e.g.*, Al^{3+}, Se^{2-}), and the molecular spinor coefficients are used as initial guesses for the 4c-CASSCF calculations. State averaging is performed on all states depicted in Fig. 4 with equal weights for each state, with the sum of weights normalized to unity. The reason to include additional states in the state averaging procedure is to avoid root-flipping problems, or to avoid biasing the CASSCF convergence (in the case of including the $^4\mathrm{S}_{3/2}$ states for group 15 elements). The states shown correspond to the lowest 6, 10, 14, 10, 6 states of the 4c-SA-CASSCF solutions for groups 13 to 17 respectively. In our DSRG calculations, full (iterative) reference relaxation is performed for all calculations.

For the calculations involving the hydroxyl radical, a complete active space of 5 electrons in 8 spinor orbitals is used, which corresponds to the $2p_\sigma$, the doubly degenerate $2p_\pi$, and the $2p_\sigma^*$ MOs. The state-averaged calculations averages over the lowest 6 states with equal weights, corresponding to the doubly degenerate X $^2\Pi_{3/2}$, $^2\Pi_{1/2}$, and A $^2\Sigma^+$ states. The default convergence criteria from Chronus Quantum are used. For all DSRG-MRPT2/3 computations, a flow parameter of 0.5 E_h^{-2} is used. The spectroscopic constants are obtained from the `psi4.diatomic.anharmonicity` function provided by the open-source package Psi4,[6] which is an implementation of the algorithm described by Bender *et al.*[7]. A grid size of 0.005 Å is used in the range of $[0.920, 1.020]$ Å, with a finer grid of 0.001 Å in the range of $[0.960, 0.990]$, for a total of 45 points, to determine the constants. The dissociation energies, D_0, are calculated using state-specific CASSCF references, as state-averaged CASSCF calculations using the same settings are not feasible due to multiple degenerate states coming in from higher (untracked) excited states.[8]

II. THE 15 'MAIN' SPLITTINGS COMPUTED WITH DIFFERENT METHODS

Splitting	Exp.	CASSCF	CASPT2	MR-CIDS+Q	MRPT2 ($s = 0.24$)	MRPT3 ($s = 0.35$)
B $^2P_{1/2} \to ^2P_{3/2}$	15.29	13.25	14.25	13.91	13.99	14.32
C $^3P_0 \to ^3P_1$	16.42	14.94	16.96	15.40	14.93	17.20
N $^2D_{5/2} \to ^2D_{3/2}$	8.71	11.09	8.16	9.40[1]	9.41	7.89
O $^3P_2 \to ^3P_1$	158.27	153.24	129.99	152.52	145.35	138.92
F $^2P_{3/2} \to ^2P_{1/2}$	404.14	382.58	380.46	388.38	384.70	391.68
Al $^2P_{1/2} \to ^2P_{3/2}$	112.06	96.81	106.60	106.96	106.70	108.35
Si $^3P_0 \to ^3P_1$	77.12	72.89	78.90	73.76	69.94	81.52
P $^2D_{3/2} \to ^2D_{5/2}$	15.61	15.34	14.01	N/A[2]	11.08	13.38
S $^3P_2 \to ^3P_1$	396.06	398.64	386.02	383.94	355.94	400.61
Cl $^2P_{3/2} \to ^2P_{1/2}$	882.35	886.86	894.86	861.80	867.69	888.44
Ga $^2P_{1/2} \to ^2P_{3/2}$	826.19	685.92	776.51	745.97	743.28	791.62
Ge $^3P_0 \to ^3P_1$	557.13	512.35	553.07	502.94[3]	485.56	570.25
As $^2D_{3/2} \to ^2D_{5/2}$	322.10	354.53	324.93	N/A[2]	227.88	327.81
Se $^3P_2 \to ^3P_1$	1989.50	1949.63	1917.35	1900.34[3]	1745.74	1991.36
Br $^2P_{3/2} \to ^2P_{1/2}$	3685.24	3683.62	3704.50	3540.14	3546.46	3683.90
MAE	–	21.2	49.0	33.4[4]	15.6	7.5
MAPE	–	7.81%	10.7%	5.60%[4]	4.98%	4.63%

[1] Two core spinors were frozen for nitrogen.

[2] The 4c-MR-CISD+Q computations were intractable for these atoms.

[3] The uncontracted cc-pVDZ basis set was used for these atoms.

[4] Unavailable data points have been omitted from averaging.

TABLE S1. Comparison between the spin-orbit splittings of the 15 second- to fourth-row p-block elements calculated with 4c-SA-CASSCF, 4c-CASPT2, 4c-MR-CISD+Q, and MRPT2 and 3 to the experimental splittings. Flow parameters, s, are in units of E_h^{-2}, and all results are reported in units of cm^{-1}, unless otherwise noted. "Exp." stands for experimental splitting, "MAE" stands for mean absolute error, and "MAPE" stands for mean absolute percentage error.

III. THE 9 ADDITIONAL SPLITTINGS NOT INCLUDED IN SUMMARY STATISTICS

Splitting	Exp.	CASSCF	CASPT2	MR-CIDS+Q	MRPT2 ($s = 0.24$)	MRPT3 ($s = 0.35$)
C $^3P_1 \rightarrow ^3P_2$	27.00	23.82	23.95	24.88	28.48	28.72
N $^4S_{3/2} \rightarrow ^2D_{5/2}$	19224.46	22910.28	20436.89	20095.77[1]	20231.34	19952.54
O $^3P_1 \rightarrow ^3P_0$	68.71	66.99	62.55	65.85	54.49	58.32
Si $^3P_1 \rightarrow ^3P_2$	146.04	138.28	133.25	140.43[3]	149.20	154.03
P $^4S_{3/2} \rightarrow ^2D_{5/2}$	11361.02	15305.18	12561.03	N/A[2]	13302.41	12666.93
S $^3P_1 \rightarrow ^3P_0$	177.59	180.62	161.92	173.46	175.36	181.66
Ge $^3P_1 \rightarrow ^3P_2$	852.83	809.65	711.39	814.20	857.09	878.13
As $^4S_{3/2} \rightarrow ^2D_{5/2}$	10592.50	14289.54	11799.71	N/A[2]	12567.66	12034.45
Se $^3P_1 \rightarrow ^3P_0$	544.86	572.88	532.84	566.74[3]	573.81	590.89
MAPE	–	13.4%	9.4%	4.5%[4]	8.5%	7.7%

[1] Two core spinors were frozen for nitrogen.

[2] The 4c-MR-CISD+Q computations were intractable for these atoms.

[3] The uncontracted cc-pVDZ basis set was used for these atoms.

[4] As the most error-prone data points were intractable for MRCI, the average is not appropriate for comparison with other methods.

TABLE S2. Comparison between additional spin-orbit splittings (those indicated with dashed arrows in Fig. 4) of the 9 second- to fourth-row p-block elements calculated with 4c-SA-CASSCF, 4c-CASPT2, 4c-MR-CISD+Q, and MRPT2 and 3 to the experimental splittings. Flow parameters, s, are in units of E_h^{-2}, and all results are reported in units of cm^{-1}, unless otherwise noted. "Exp." stands for experimental splitting, and "MAPE" stands for mean absolute percentage error.

IV. SUMMARY STATISTICS OVER THE MAIN 15 SPIN-ORBIT SPLITTINGS

Method		MSE / cm^{-1}	MAE / cm^{-1}	MSPE / %	MAPE / %
CASSCF		-16.130	21.115	-2.744	7.804
CASPT2		-49.168	49.261	-9.704	10.771
DSRG-MRPT2	$s = 0.5$	-37.949	38.392	-7.546	8.958
	$s = 0.35$	-27.853	28.503	-6.043	7.081
	$s = 0.2$	-14.024	17.891	-3.556	5.370
DSRG-MRPT3	$s = 0.5$	-10.581	13.636	-3.582	5.758
	$s = 0.35$	-2.596	7.465	-2.428	4.632
	$s = 0.2$	7.620	18.941	-0.787	5.305

TABLE S3. The summary error statistics for the selected four-component methods over second- to fourth-row p-block elements.

V. SUMMARY STATISTICS BROKEN DOWN INTO GROUPS

Method		MAE	13	14	15	16	17
CASSCF		abs.	52.523	16.831	11.692	15.827	8.702
		pct.	14.647	7.511	13.011	1.945	1.903
CASPT2		abs.	29.856	26.745	33.149	98.929	57.628
		pct.	7.768	10.403	22.090	10.180	3.413
DSRG-MRPT2	$s = 0.5$	abs.	28.663	6.031	9.000	103.224	45.041
		pct.	7.205	4.413	14.377	15.482	3.314
	$s = 0.35$	abs.	30.294	5.763	4.788	71.755	29.917
		pct.	7.538	3.317	9.892	11.943	2.716
	$s = 0.2$	abs.	34.017	6.053	3.954	32.046	13.385
		pct.	8.399	3.929	5.062	7.015	2.443
DSRG-MRPT3	$s = 0.5$	abs.	11.525	7.638	2.363	29.064	17.591
		pct.	4.155	5.441	11.554	6.391	1.250
	$s = 0.35$	abs.	13.084	6.101	2.924	8.589	6.627
		pct.	4.614	4.273	1.270	4.489	2.716
	$s = 0.2$	abs.	17.266	3.785	9.087	30.749	33.818
		pct.	5.687	2.600	9.726	5.788	2.722

TABLE S4. Summary error statistics for selected four-component methods across groups 13 through 17. The mean absolute errors (abs.) are in cm^{-1}, and the mean absolute percentage errors (pct.) are in percentage points.

VI. SUMMARY STATISTICS BROKEN DOWN INTO ROWS

Method		MSE / cm^{-1}	MAE / cm^{-1}	MSPE / %	MAPE / %
		B-F			
CASSCF		-5.548	6.496	-0.730	11.622
CASPT2		-6.889	7.167	-4.503	7.702
DSRG-MRPT2	$s = 0.5$	-12.771	13.295	-12.258	15.451
	$s = 0.35$	-11.796	12.144	-9.343	11.462
	$s = 0.2$	-9.812	10.339	-4.532	7.742
DSRG-MRPT3	$s = 0.5$	-6.721	7.125	-8.160	10.623
	$s = 0.35$	-6.564	6.876	-5.272	7.170
	$s = 0.2$	-6.137	6.541	-2.119	5.763
		Al-Cl			
CASSCF		-4.019	5.052	-4.101	4.362
CASPT2		-14.369	14.369	-10.980	10.980
DSRG-MRPT2	$s = 0.5$	-12.146	12.950	-3.630	4.673
	$s = 0.35$	-5.983	8.395	-3.631	4.627
	$s = 0.2$	-0.308	5.774	-3.304	4.238
DSRG-MRPT3	$s = 0.5$	-2.181	4.413	-1.425	4.320
	$s = 0.35$	1.820	4.199	-2.014	5.037
	$s = 0.2$	7.387	10.685	-1.877	6.340
		Ga-Br			
CASSCF		-38.823	51.797	-3.399	7.427
CASPT2		-126.248	126.248	-13.630	13.630
DSRG-MRPT2	$s = 0.5$	-88.930	88.930	-6.751	6.751
	$s = 0.35$	-64.971	64.971	-5.154	5.154
	$s = 0.2$	-31.952	37.560	-2.832	4.130
DSRG-MRPT3	$s = 0.5$	-22.842	29.371	-1.160	2.332
	$s = 0.35$	-3.043	11.320	0.000	1.688
	$s = 0.2$	21.609	39.597	1.633	3.811

TABLE S5. Summary statistics of selected four-component methods for each row. Mean signed and average errors (MSE and MAE) and mean signed and average percentage errors (MSPE and MAPE) are reported.

VII. RESULTS FOR USING THE AUGMENTED CC-PVTZ BASIS

We also augmented other second row elements with a p function each with exponents determined by scaling the fluorine exponent by the ratio between the exponents of the tightest p functions in the original basis set of a given element and fluorine, resulting in exponents of 68.8243, 106.9535, 152.2273, and 196.9866 for boron, carbon, nitrogen, and oxygen respectively.

Splitting	Exp.	CASSCF orig.	CASSCF aug.	MRPT2 orig. ($s = 0.24$)	MRPT2 aug. ($s = 0.35$)	MRPT3 orig. ($s = 0.24$)	MRPT3 aug. ($s = 0.35$)
B $^2P_{1/2} \rightarrow {}^2P_{3/2}$	15.29	13.25	13.77	13.99	14.81	14.32	14.88
C $^3P_0 \rightarrow {}^3P_1$	16.42	14.94	15.42	14.93	17.51	17.20	17.75
C $^3P_1 \rightarrow {}^3P_2$	27.00	23.82	24.77	28.48	29.56	28.72	29.81
N $^2D_{5/2} \rightarrow {}^2D_{3/2}$	8.71	11.09	11.01	9.41	8.02	7.89	7.73
N $^4S_{3/2} \rightarrow {}^2D_{5/2}$	19224.46	22910.28	22914.03	20231.34	20235.14	19952.54	19956.74
O $^3P_2 \rightarrow {}^3P_1$	158.27	153.23	157.34	145.35	133.37	138.92	142.57
O $^3P_1 \rightarrow {}^3P_0$	68.71	66.99	68.95	54.49	56.12	58.32	60.07
F $^2P_{3/2} \rightarrow {}^2P_{1/2}$	404.14	382.58	393.36	384.70	391.15	391.68	403.10

TABLE S6. Splittings in all 8 calculated spin-orbit splittings for second-row elements before and after augmenting the cc-pVTZ basis with a tight p function. Flow parameters are in units of E_h^{-2}, 'orig.' stands for 'original basis', and 'aug.' stands for 'augmented basis'. All results are in cm^{-1}.

Splitting	CASSCF	MRPT2 ($s = 0.24$)	MRPT3 ($s = 0.35$)
B $^2P_{1/2} \to ^2P_{3/2}$	3.40	5.95	3.78
C $^3P_0 \to ^3P_1$	2.94	16.73	3.14
C $^3P_1 \to ^3P_2$	3.53	4.36	3.69
N $^2D_{5/2} \to ^2D_{3/2}$	-0.87	-12.62	-2.00
N $^4S_{3/2} \to ^2D_{5/2}$	0.02	0.02	0.02
O $^3P_2 \to ^3P_1$	2.59	-7.61	2.74
O $^3P_1 \to ^3P_0$	2.85	2.36	3.12
F $^2P_{3/2} \to ^2P_{1/2}$	2.67	1.64	2.92
Avg. improvement	2.14	1.35	2.18
MAPE (orig.)	11.44	8.74	7.64
MAPE (aug.)	9.18	8.72	7.38

TABLE S7. Percentage error improvements in all 8 calculated spin-orbit splittings for second-row elements after augmenting the cc-pVTZ basis with a tight p function. A positive percentage signifies that the result after the augmentation is closer to the experimental splitting, and *vice versa* for a negative percentage. Flow parameters are in units of E_h^{-2}, "Avg." stands for "average", "MAPE" stands for mean absolute percentage error, "orig." stands for "original basis", and "aug." stands for "augmented basis". All results are in percentage points.

VIII. RESULTS FOR CALCULATIONS USING DC COEFFICIENTS

We have experimented with using only the DC Hamiltonian for the 4c-CASSCF stage, using the resulting coefficients to transform the DCB atomic spinor integrals, which are then fed into 4c-DSRG-MRPT2. We can see that the performance is very similar to those using full DCB molecular spinor coefficients. This demonstrates that iterative reference relaxation can largely correct for inexact starting CI and MO coefficients, and can therefore be used to bypass expensive iterative DCB integral transformations in the SCF stages. This is further supported by the fact that, if the same MO coefficients used in the AO to MO transformation using DC atomic spinor integrals ("0.2 DCDC"), the resulting error distribution deteriorates significantly.

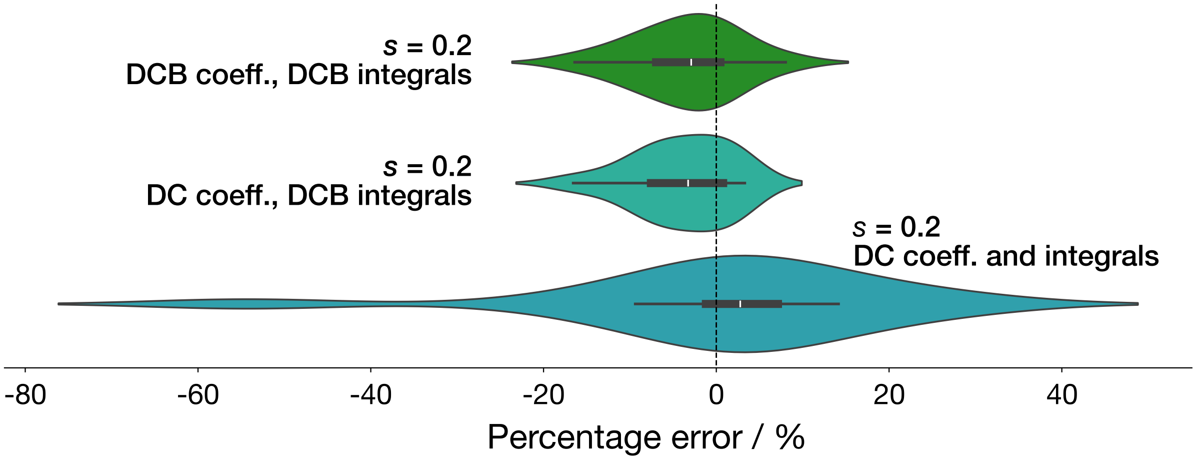

FIG. S1. Distributions of the splitting errors for $s = 0.2\ E_{\mathrm{h}}^{-2}$, using DCB-CASSCF molecular spinor coefficients (green) or DC-CASSCF coefficients (teal) with DCB atomic spinor integrals, and using DC-CASSCF coefficients with DC atomic spinor integrals (blue)

Splitting	Exp.	0.2	0.2 DC	0.2 DCDC
B $^2P_{1/2} \rightarrow ^2P_{3/2}$	15.29	14.25	13.99	19.42
C $^3P_0 \rightarrow ^3P_1$	16.42	17.73	16.85	20.15
N $^2D_{5/2} \rightarrow ^2D_{3/2}$	8.71	8.56	8.33	3.98
O $^3P_2 \rightarrow ^3P_1$	158.27	132.32	132.05	133.39
F $^2P_{3/2} \rightarrow ^2P_{1/2}$	404.14	380.90	380.39	423.01
Al $^2P_{1/2} \rightarrow ^2P_{3/2}$	112.06	104.01	104.00	110.79
Si $^3P_0 \rightarrow ^3P_1$	77.12	77.78	78.27	82.35
P $^2D_{3/2} \rightarrow ^2D_{5/2}$	15.61	14.01	14.37	17.82
S $^3P_2 \rightarrow ^3P_1$	396.06	390.51	391.87	407.04
Cl $^2P_{3/2} \rightarrow ^2P_{1/2}$	882.35	895.35	895.35	932.38
Ga $^2P_{1/2} \rightarrow ^2P_{3/2}$	826.19	733.23	729.89	748.70
Ge $^3P_0 \rightarrow ^3P_1$	557.13	540.96	540.82	553.96
As $^2D_{3/2} \rightarrow ^2D_{5/2}$	322.10	332.20	332.72	346.24
Se $^3P_2 \rightarrow ^3P_1$	1989.50	1924.86	1924.60	1963.86
Br $^2P_{3/2} \rightarrow ^2P_{1/2}$	3685.24	3689.16	3689.57	3759.85
MAE	–	17.9	18.2	22.1
MAPE	–	5.4%	5.2%	11.7%

TABLE S8. Comparison between the spin-orbit splittings of the 15 second- to fourth-row p-block elements calculated with MRPT2 with standard DCB Hamiltonian ('0.2'), MRPT2 with MO coefficients from a DC-CASSCF calculation transformed with DCB integrals ('0.2 DC'), and MRPT2 with MO coefficient from a DC-CASSCF calculation transformed with DC integrals ('0.2 DCDC') to the experimental splittings. All calculations employed a flow parameter, s, of 0.2 E_h^{-2}, and all results are reported in units of cm^{-1}, unless otherwise noted. 'Exp.' stands for experimental splitting, 'MAE' stands for mean absolute error, and 'MAPE' stands for mean absolute percentage error.

IX. RESULTS SHOWING THE EFFECT OF ORBITAL RELAXATION

Our theory can straightforwardly incorporate the effect of orbital relaxation by iteratively re-diagonalizing the active space effective Hamiltonian in the CAS. The effect of this can be quantified in Fig. S2. The data used to generate this figure can be found in Table S9.

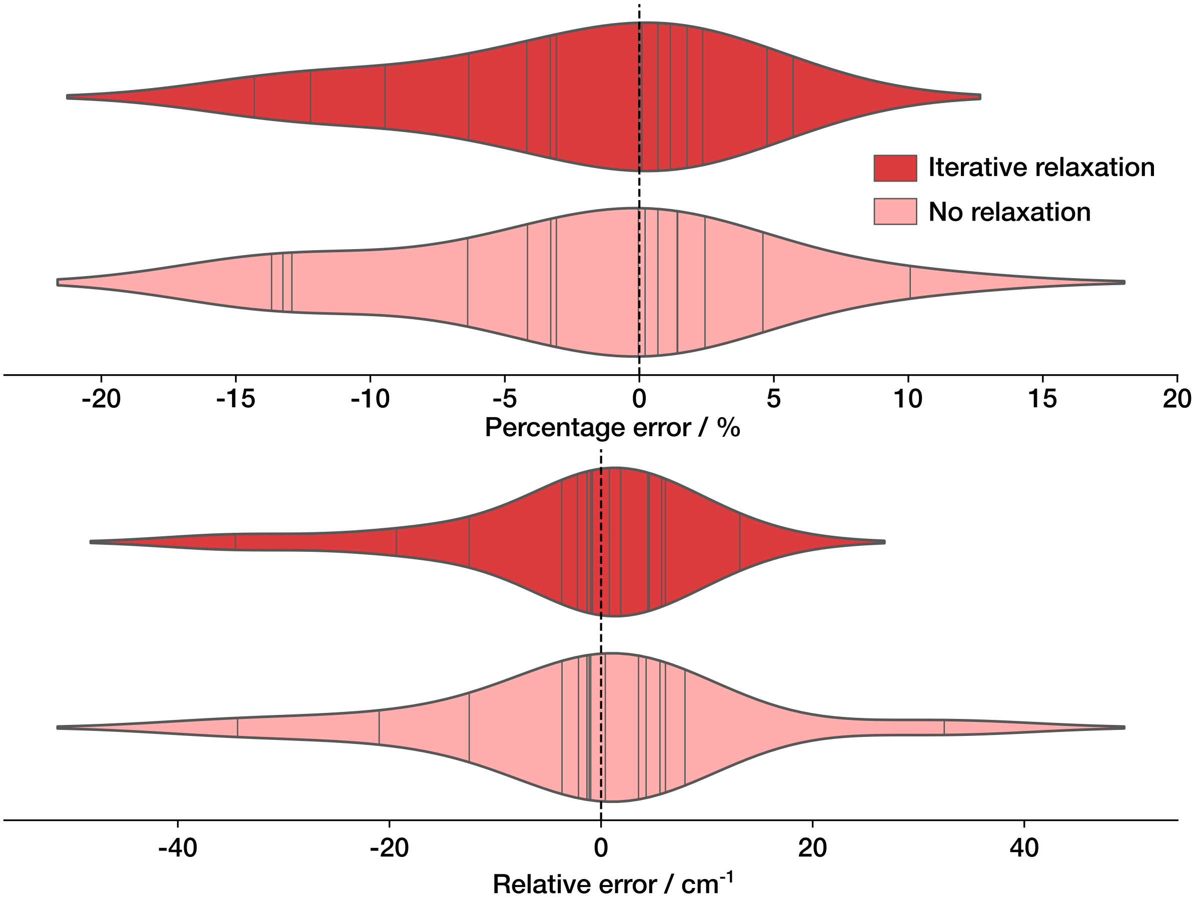

FIG. S2. Distributions of the splitting errors for $s = 0.35\ E_{\mathrm{h}}^{-2}$ at the 4c-DSRG-MRPT3 level of theory. The iteratively relaxed results (deep red) show a more compact error distribution around the experimental values compared to the unrelaxed results (light red).

Splitting	Exp.	Relaxed	Unrelaxed
B $^2P_{1/2} \rightarrow ^2P_{3/2}$	15.29	14.32	14.31
C $^3P_0 \rightarrow ^3P_1$	16.42	17.20	16.82
N $^2D_{5/2} \rightarrow ^2D_{3/2}$	8.71	7.89	7.59
O $^3P_2 \rightarrow ^3P_1$	158.27	138.92	137.29
F $^2P_{3/2} \rightarrow ^2P_{1/2}$	404.14	391.68	391.68
Al $^2P_{1/2} \rightarrow ^2P_{3/2}$	112.06	108.35	108.37
Si $^3P_0 \rightarrow ^3P_1$	77.12	81.52	80.66
P $^2D_{3/2} \rightarrow ^2D_{5/2}$	15.61	13.38	13.48
S $^3P_2 \rightarrow ^3P_1$	396.06	400.61	401.62
Cl $^2P_{3/2} \rightarrow ^2P_{1/2}$	882.35	888.44	888.44
Ga $^2P_{1/2} \rightarrow ^2P_{3/2}$	826.19	791.62	791.84
Ge $^3P_0 \rightarrow ^3P_1$	557.13	570.25	565.07
As $^2D_{3/2} \rightarrow ^2D_{5/2}$	322.10	327.81	354.53
Se $^3P_2 \rightarrow ^3P_1$	1989.50	1991.36	1993.76
Br $^2P_{3/2} \rightarrow ^2P_{1/2}$	3685.24	3683.90	3683.90
MAE	–	7.46	9.15
MAPE	–	4.6%	5.2%

TABLE S9. Comparison between the spin-orbit splittings of the 15 second- to fourth-row p-block elements calculated with MRPT3 with iterative reference relaxation ('Relaxed') or without any relaxation ('Unrelaxed'). All calculations employed a flow parameter, s, of 0.35 E_h^{-2}, and all results are reported in units of cm^{-1}, unless otherwise noted. 'Exp.' stands for experimental splitting, 'MAE' stands for mean absolute error, and 'MAPE' stands for mean absolute percentage error.

REFERENCES

[1] T. H. Dunning, J. Chem. Phys. **90**, 1007 (1989).

[2] D. E. Woon and T. H. Dunning, Jr., J. Chem. Phys. **98**, 1358 (1993).

[3] A. K. Wilson, D. E. Woon, K. A. Peterson, and T. H. Dunning, Jr., J. Chem. Phys. **110**, 7667 (1999).

[4] B. Zhang, J. E. Vandezande, R. D. Reynolds, and H. F. Schaefer III, J. Chem. Theory Comput. **14**, 1235 (2018).

[5] L. Visscher and K. G. Dyall, At. Data Nucl. Data Tables **67**, 207 (1997).

[6] D. G. A. Smith, L. A. Burns, A. C. Simmonett, R. M. Parrish, M. C. Schieber, R. Galvelis, P. Kraus, H. Kruse, R. Di Remigio, A. Alenaizan, A. M. James, S. Lehtola, J. P. Misiewicz, M. Scheurer, R. A. Shaw, J. B. Schriber, Y. Xie, Z. L. Glick, D. A. Sirianni, J. S. O'Brien, J. M. Waldrop, A. Kumar, E. G. Hohenstein, B. P. Pritchard, B. R. Brooks, H. F. Schaefer III, A. Y. Sokolov, K. Patkowski, A. E. DePrince III, U. Bozkaya, R. A. King, F. A. Evangelista, J. M. Turney, T. D. Crawford, and C. D. Sherrill, J. Chem. Phys. **152**, 184108 (2020).

[7] J. D. Bender, S. Doraiswamy, D. G. Truhlar, and G. V. Candler, J. Chem. Phys. **140**, 054302 (2014).

[8] I. Easson and M. H. L. Pryce, Can. J. Phys. **51**, 518 (1973).
